# Nutrient-Dependent Changes of Protein Palmitoylation: Impact on Nuclear Enzymes and Regulation of Gene Expression

**DOI:** 10.3390/ijms19123820

**Published:** 2018-11-30

**Authors:** Matteo Spinelli, Salvatore Fusco, Claudio Grassi

**Affiliations:** 1 Institute of Human Physiology, Università Cattolica del Sacro Cuore, Rome 00168, Italy; matteo.spinelli@unicatt.it; 2 Fondazione Policlinico Universitario A. Gemelli IRCSS, Rome 00168, Italy

**Keywords:** protein palmitoylation, nutrient-dependent signals, chromatin remodelers, transcription factors, epigenetics, personalized medicine

## Abstract

Diet is the main environmental stimulus chronically impinging on the organism throughout the entire life. Nutrients impact cells via a plethora of mechanisms including the regulation of both protein post-translational modifications and gene expression. Palmitoylation is the most-studied protein lipidation, which consists of the attachment of a molecule of palmitic acid to residues of proteins. *S*-palmitoylation is a reversible cysteine modification finely regulated by palmitoyl-transferases and acyl-thioesterases that is involved in the regulation of protein trafficking and activity. Recently, several studies have demonstrated that diet-dependent molecules such as insulin and fatty acids may affect protein palmitoylation. Here, we examine the role of protein palmitoylation on the regulation of gene expression focusing on the impact of this modification on the activity of chromatin remodeler enzymes, transcription factors, and nuclear proteins. We also discuss how this physiological phenomenon may represent a pivotal mechanism underlying the impact of diet and nutrient-dependent signals on human diseases.

## 1. Introduction

It is widely established that diet- and nutrient-related signals impinge on cell physiology and play a pivotal role in the health/disease balance of human bodies [1]. Diet-derived molecules such as insulin, glucose, and fatty acids (FA) influence cell signaling and regulate both enzyme activity and gene expression through a plethora of only partially known mechanisms [2,3,4].

**Figure 1:**
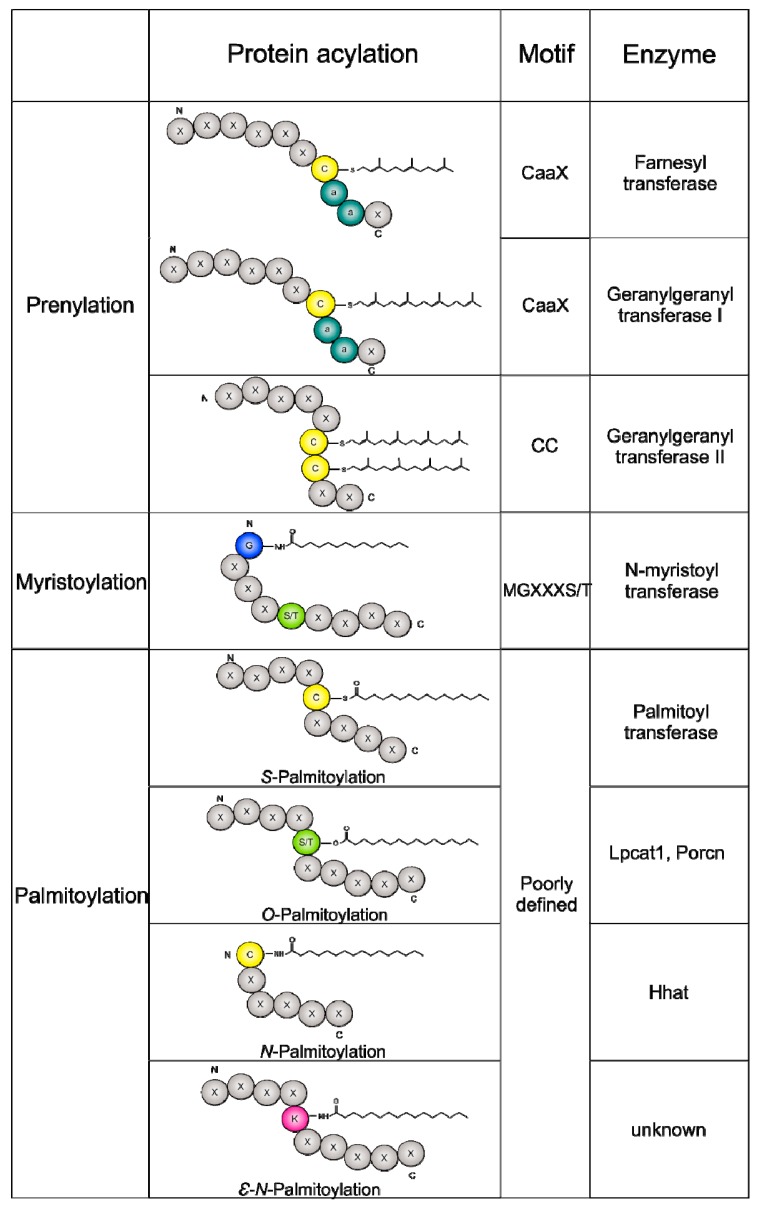
Lipid modifications. Prenylation, myristoylation, and palmitoylation are the most relevant protein lipidation processes regulating the interaction of modified proteins with cellular membranes. Protein prenylation involves the transfer of either a farnesyl or a geranylgeranyl moiety to the C-terminal cysteine(s) (C, yellow) of the target protein. Myristoylation occurs on an N-terminal glycine (G, blue), while palmitoylation may target different amino acids including cysteine, serine (S, green), or lysine (K, violet). a, aliphatic amino acid; M, methionine; X, undefined amino acid; T, threonine.

Carbohydrates and lipids are the main energy sources for cells and their catabolism produces metabolic intermediates such as adenosine triphosphate (ATP) and acetyl-CoA, which are also fundamental donor molecules for protein phosphorylation and acetylation, respectively [5,6]. Moreover, FA represent the substrate for protein acylation (also named protein lipidation), a group of post-translational modifications (PTMs) including myristoylation, prenylation, and palmitoylation (Figure 1). Protein lipidation dynamically regulates cell response to extrinsic and intrinsic environmental cues [7,8], modulating protein stability and interaction, enzymatic activities, and protein localization [9,10,11,12,13].

In the following sections, we first summarize how FA and nutrient-dependent signals regulate protein palmitoylation. Then, we discuss the mechanisms underlying gene expression modulation by protein palmitoylation, with particular focus on nuclear proteins such as chromatin remodeler enzymes, transcription factors, and histone proteins. An increasing body of evidence from experimental models highlights the role of environmental epigenetics in disease susceptibility [14]. An intriguing hypothesis is that aberrant palmitoylation might represent a mechanism underlying the effects of metabolic stress on human diseases. The role of protein palmitoylation in cancer and neurological diseases will be also discussed.

## 2. Biochemistry of Palmitoylation and Biological Functions

Protein acylation consists in the binding of saturated or unsaturated FA to glycine, serine, lysine, or cysteine residues of proteins. The number of carbons in the fatty acid substrate determines the kind of fatty acylation. The main protein acylations occurring in cells are myristoylation, prenylation, and palmitoylation.

Myristoylation consists in the attachment of a molecule of myristate, a 14-carbon saturated fatty acid, to N-terminal glycine residues via an irreversible amide linkage, catalyzed by the ubiquitous eukaryotic enzyme *N*-myristoyltransferase (NMT) [15]. Point mutation experiments performed in vitro on synthetic peptides resembling the N-terminal sequences of known *N*-myristoylated proteins allowed the identification of the putative amino acid motif targeted by myristoylation (Met–Gly–X–X–X–Ser/Thr) [16]. Recent studies suggested that many proteins are myristoylated in human cells [17]. Typically, myristoylation facilitates the protein–membrane association that is required for targeting proteins in specific organelles for their biological function.

Prenylation involves the addition of prenyl groups via a thioether bond to a cysteine residue and is commonly considered an irreversible PTM [18]. Most prenylated proteins contain a conserved CAAX motif at the C-terminus (C: cysteine; A: aliphatic amino acid; X: any amino acid), where the consensus cysteine residue can be modified by the addition of farnesyl (15-carbon) or geranylgeranyl (20-carbon) groups by two different enzymes, farnesyltransferase or geranylgeranyltransferase I, respectively [19] (Figure 1). About 2% of eukaryotic proteins are supposed to be prenylated [20]. Interestingly, prenylation increments the hydrophobicity of the C-terminus of proteins, increasing their capacity to interact with other proteins and cellular membranes [21,22].

Palmitoylation is the most studied fatty acylation, consisting in the attachment of a palmitic acid (PA) molecule, a 16-carbon saturated FA, to proteins. Palmitoylation is the only reversible fatty acylation finely modulated by a class of enzymes named palmitoyl acyltransferases (PATs) [23].

About 10% of the genome codifies for palmitoylable proteins, which all together constitute the so-called “palmitoylome”. High-throughput screening using mass spectrometry has revealed that human diseases including cancer, viral infections, and immunological and neurodegenerative disorders are associated with severe alterations in the palmitoylome [24]. Moreover, recent studies have demonstrated that nutrient-derived molecules such as insulin and fatty acids may critically change protein palmitoylation [25,26].

Palmitoylation was first studied for its impact on protein trafficking between the plasma membrane and the cytosol (or Golgi apparatus) but, over the years, several nuclear proteins have been identified as targets of palmitoylation, suggesting a potential role of this acylation in the control of gene expression.

**Figure 2:**
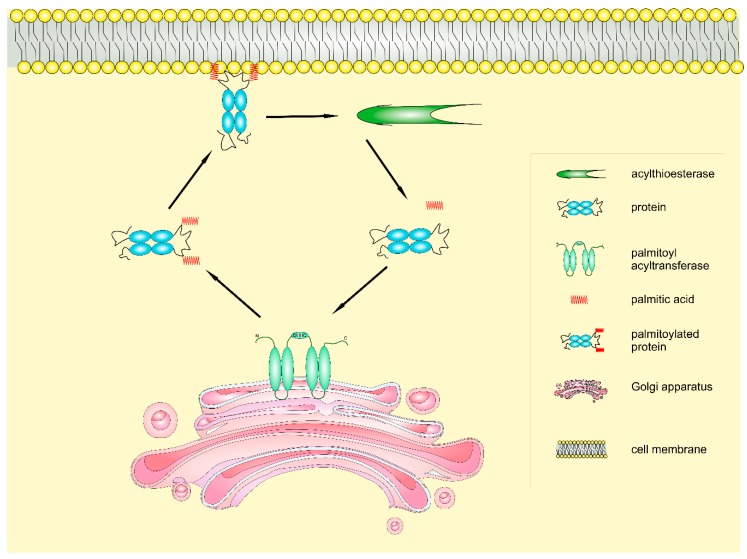
Protein *S*-palmitoylation biochemistry. Protein *S*-palmitoylation is a reversible modification dynamically regulated by palmitoyl acyltransferases, which add a molecule of palmitic acid to proteins, and acylthioesterases, which catalyze protein deacylation by removing the acyl moiety.

Palmitoylation targets several proteins residues, including cysteine (*S*-palmitoylation or *N*-palmitoylation when it occurs at the N-terminus of the protein) and serine (*O*-palmitoylation), as illustrated in Figure 1. The most studied enzymatic reaction involving PA is *S*-palmitoylation, in which PA is reversibly linked to cysteine residues using palmitoyl coenzyme A as a substrate [27] (Figure 2).

Conversely, both *O*-palmitoylation and *N*-palmitoylation appear to be irreversible [28,29,30]. Finally, the epsilon-amino group of lysine has also been proposed as a possible target of palmitoylation [31].

PATs are characterized by four to six transmembrane domains and by the presence of a 51-amino acid zinc finger-like domain including an aspartate-histidine-histidine-cysteine (DHHC) motif [32]. The highly conserved DHHC domain is inside the catalytic domain and mutations of this motif box impair the palmitoyl-transfer reaction [33].

To date, seven PATs (also named DHHC proteins) expressed in yeast [34,35] and 23 DHHC in mammalian cells have been discovered [36].

The biochemistry of palmytoilation is still not sufficiently understood, but it has been demonstrated that PAT enzymes catalyze the addition of palmitate to proteins via a two-step mechanism. First, they bind and hydrolyze the palmitoyl-CoA, forming a chemical intermediate between enzyme and palmitate. Subsequently, the acyl group is transferred to the target protein [32,37].

On the other side, acyl protein thioesterases catalyze the thioester hydrolysis of palmitoylated cysteines. These enzymes are usually localized in two cellular compartments: lysosomes, as the Palmitoyl-Protein Thioesterase 1 (PPT1), or the cytosol, such as acyl-protein thioesterase 1 (APT1) and 2 (APT2) [38,39]. Recently, the α/β-Hydrolase domain-containing protein 17 (ABHD17) family has been shown to remove palmitate from NRAS [40]. Notably, APT1 and APT2 inhibition reduces palmitate turnover on some proteins but has no effect on NRAS or postsynaptic density (PSD)-95. Similarly, the overexpression of ABHD16A, ABHD6, or APT1/2 had a weak effect on NRAS and PSD-95 depalmitoylation. However, the expression of ABHD17A, ABHD17B, or ABHD17C accelerated the hydrolysis of palmitate. These data have also been confirmed by ABHD17A/B/C knockdown in human embryonic kidney 293T cells since NRAS palmitate turnover was strongly inhibited when all three ABHD17 proteins were simultaneously silenced [40].

Cycles of protein palmitoylation/depalmitoylation are essential for the modulation of many aspects of cell physiology. Moreover, the development of click chemistry-based FA probes has revealed the dynamic processes underlying several signaling pathways [41,42]. For instance, palmitoylation of the tyrosine kinase Lck induces the recruitment of the enzyme to the plasma membrane and is essential for the activation of T cell receptor signaling in human T lymphocytes [43]. Furthermore, the glutamate receptor activation-dependent turnover of PSD-95 palmitoylation is fundamental for the modulation of synaptic strength and plasticity in neurons [44].

Cycles of acylation and deacylation are also required for the plasma membrane association of RAS-family and guanine nucleotide-binding (G) proteins. The pharmacological inhibition of thioesterases is able to block RAS trafficking and repress RAS oncogenic activity [45].

Actually, the *S*-palmitoylated proteins lack a specific sequence motif. This peculiarity makes it difficult to predict which proteins will undergo this PTM and, therefore, requires empirical identification. In this regard, the proteomic approach has highlighted an increasing number of identified palmitoylated proteins, opening up many exciting avenues for research [46].

## 3. Nutrient-Dependent Regulation of Protein Palmitoylation

Diet-derived molecules can impact cell physiology through multiple mechanisms including the regulation of both protein PTM and gene expression [1,3,4]. Recently, several studies have highlighted how nutrient-dependent signals may influence metabolism by changing protein palmitoylation in several tissues. Insulin is the most important hormone controlling both carbohydrate and lipid metabolism. The main mechanism by which insulin regulates glucose uptake and energy storage involves the exposure of glucose transporter (GLUT) 4 on the plasma membrane surface [47]. The cellular localization of GLUT4 is governed by a finely regulated process of vesicle recycling [48]. This glucose transporter is predominantly expressed in adipose and muscle tissues and plays a critical role in the maintenance of glucose homeostasis and peripheral insulin sensitivity [49]. During fasting, GLUT4 is mainly maintained in intracellular compartments, but postprandial insulin stimulation promotes its translocation to the plasma membrane via the palmitoylation of cysteine 233 [50]. Accordingly, the deletion of DHHC7, the main PAT involved in GLUT4 palmitoylation, induces hyperglycemia and glucose intolerance in mice [51]. More generally, insulin can affect the palmitoylation of more than 300 proteins in human endothelial cells and defects of palmitoylation affect endothelial cell migration [52].

Proteomic analyses in adipocytes revealed a wide range of palmitoylated proteins including those involved in GLUT4 membrane translocation (e.g., insulin-regulated aminopeptidase) and the Janus kinase/signal transducers and activators of transcription (JAK/STAT) pathway (e.g., JAK1 and 2; STAT1, 3, and 5A; Src homology- 2 domain containing phosphatase) [53]. This study also showed that the palmitoylation of GLUT4, IRAP, and JAK1 plays a regulatory role in adipose function and is impaired in obesity and insulin resistance.

In line with these observations, we demonstrated that feeding mice with a high-fat diet (HFD) increased the deposition of PA and altered insulin signaling in the hippocampus [26]. The insulin desensitization in the brain led to DHHC3 overexpression via FoxO3a activation that, together with PA accumulation, induced the hyper-palmitoylation of α-amino-3-hydroxy-5-methyl-4-isoxazolepropionic acid (AMPA) glutamate receptor subunit GluA1. These molecular changes were responsible for the synaptic plasticity impairment and cognitive deficits observed in HFD-fed mice. The critical role of aberrant GluA1 palmitoylation in the HFD-related alteration of synaptic plasticity was confirmed through the overexpression of palmitoylation-deficient GluA1 and the hippocampus-specific silencing of *Zdhhc3*. Both experimental approaches prevented the impairment of synaptic plasticity and cognitive deficits in HFD-fed mice. Interestingly, the intranasal delivery of the palmitoyltransferase inhibitor 2-Bromopalmitate (2-BP) restored normal levels of GluA1 palmitoylation in the hippocampus and counteracted the impairment of learning and memory in HFD-fed mice [26]. This was the first evidence that the alteration of insulin signaling may impinge on the palmitoylation of neuronal proteins. Moreover, our findings revealed that preventing aberrant protein palmitoylation may counteract the brain insulin resistance-dependent impairment of synaptic plasticity.

The tight relationship between lipid metabolism and protein palmitoylation is also indicated by the high *S*-depalmitoylation activity detected in the mitochondria of mammalian cells [54]. These findings confirm that palmitoylation may occur in cellular compartments other than the cytosol and Golgi apparatus and suggest a role of this PTM in the regulation of mitochondrial lipid homeostasis. Accordingly, it has been demonstrated that the palmitoylation of the FA translocase CD36 modulates both its localization and activity [55]. Moreover, increased CD36 palmitoylation was observed in livers of mice with non-alcoholic fatty liver disease (NASH) [53] and was directly related to intracellular lipid accumulation in human liver biopsies obtained from patients with NASH [56].

Finally, protein palmitoylation is also modulated by glucose concentration in rat pancreatic islets [57]. Specifically, protein *S*-depalmitoylation is involved in glucose-dependent insulin secretion in β-cells [58]. Accordingly, signaling pathways inducing insulin secretion in β-cells involve numerous protein targets for acylation including receptors [59], ion channels [60,61], soluble N-ethylmaleimide-sensitive factor attachment protein receptor (SNARE) proteins [62], and G-proteins [63].

In summary, nutrient-related molecules may affect the palmitoylation of numerous proteins in different organs and cell types. Palmitoylated proteins have been mainly described in the cytosol and Golgi apparatus, although several targets have been identified in all intracellular organelles. Therefore, protein palmitoylation may represent a novel signal transduction mechanism underlying the effect of metabolic stimuli on cell physiology. Considering the pivotal role of this post-translational modification in regulating both the localization and activity of targeted proteins, emerging interest is turning to the palmitoylation of nuclear proteins in the regulation of gene expression.

## 4. Palmitoylation of Transcription Factors

Lipid modifications increase the proteins’ hydrophobicity and thus their tendency to associate with membranes. This may facilitate the proteins’ propensity to anchor any membranes and, in the case of transcription factors, to reach the nucleus.

This scenario occurs for all sex steroid receptors (SR), such as estrogen, progesterone, and androgen receptors, which have a highly conserved palmitoylation motif of nine amino acids [64]. Estrogen receptor (ER) α, the most characterized among the palmitoylated SR, translocates from the plasma membrane to the nucleus upon de-*S*-palmitoylation [65]. Proteomic studies showed that the heat shock protein 27 (Hsp27) impinges on ERα palmitoylation by promoting its interaction with the acyl transferase DHHC7 and/or DHHC21 [66]. The palmitoylation of ERα induces its binding to Caveolin-1 [67], followed by localization in lipid rafts [66] and the activation of steroid signaling leading to DNA transcription [68]. The critical role of palmitoylation in steroid signaling has been demonstrated by the transgenic mouse model of palmitoylation-deficient ERα. These animals showed fertility impairment, veins vasodilation, and endothelial repair due to the loss of receptor localization to the membrane and diminished estrogen pathway activation [69].

Recent studies have also identified the TEA domain transcription factor family (TEAD1-4) as targets of *S*-palmitoylation [70]. TEAD is known as a transcriptional effector of the Hippo signaling pathway, which is involved in tissue homeostasis, proliferation, and cell growth. TEADs contain three evolutionarily conserved cysteine residues (C53S, C327S, and C359S), which are palmitoylated without affecting the localization of the transcription factors [71]. Crystallography revealed that the palmitoyl group is located inside a hydrophobic pocket of TEAD2 [71]. Palmitoylation regulates the protein stability of TEADs, facilitating their interaction with the transcription co-activators Yes-associated protein (YAP) and Tafazzin (TAZ) [71]. Once activated, TEADs co-localize with YAP/TAZ in the nucleus. ChiP-seq experiments pointed out that both transcription factors co-localize on the enhancer regions of DNA [72].

Moreover, the palmitoylation of TEADs on three cysteine residues occurs in vitro even in PAT-free conditions, suggesting that these transcription factors may undergo enzyme-independent autopalmitoylation [70,73]. However, in vivo TEAD palmitoylation may require specific enzymes because bacterially expressed TEAD is not efficiently palmitoylated [71]. A more in-depth knowledge of the functional role of TEAD palmitoylation would be very important considering the implications of the Hippo signaling pathway in cell proliferation and cancer.

The palmitoylation of transcription factors also plays a critical role in stress-dependent gene activation [73]. In plants, the palmitoylation of the transcription factor MfNACsa, a member of the NAM, ATAF1/2, and CUC2 (NAC) family, regulates its localization and is part of an orchestrated signaling cascade leading to drought tolerance. In particular, *S*-palmitoylation anchors MfNACsa to the plasma membrane, whereas under drought stress MfNACsa is de-*S*-palmitoyled by the thioesterase APT1 and translocates to the nucleus [74]. Similarly, the fatty acylation of nuclear factor of activated T cells 5α (NFAT5a) affects its nuclear import and modulates high salt stress-mediated transcriptional activity in mammals [75]. Since changes of protein palmitoylation may occur in response to metabolic stress [76], it would be interesting to deepen the knowledge of the transcription factors whose localization and interaction with the regulatory sequences can be influenced by this post-translational modification.

## 5. Palmitoylation of Chromatin Remodelers and Histone Proteins

Gene expression is finely modulated through both the transcription factor activity and the chromatin organization [77]. Chromatin remodelers (e.g., enzymes regulating acetylation and/or methylation of histone proteins) influence the assembly of transcriptional machinery by inducing histone post-translational modifications changing the DNA architecture [78]. Moreover, chromatin remodelers undergo enzymatic modifications regulating their activity and localization [79]. The *S*-palmitoylation of histone acetyltransferase p300 is required for its accumulation at the nuclear compartment, since cell treatment with 2-BP inhibits the differentiation of neuronal cells by blocking the nuclear import of p300 [80].

The switch from self-renewal to the differentiation of neural stem cells is also modulated by the *S*-palmitoylation of the adenovirus early region 1A (E1A)-like inhibitor of differentiation 1 (EID1). Indeed, the inhibition of EID1 palmitoylation reduces its proteasome-dependent degradation and promotes its interaction with CREB-binding protein (CBP)/p300 complex, leading to decreased histone acetyltransferases activity and the suppression of differentiation genes [81].

Recently, it has been demonstrated that sirtuins, which are members of a highly conserved family of nicotinamide adenine dinucleotide (NAD^+^)-dependent protein deacetylases, catalyze the hydrolysis of long-chain fatty acyl groups from the lysine residues of proteins [82,83]. The first sirtuin discovered with the ability of removing long-chain fatty acyl groups was *Plasmodium falciparum* Sir2A (PfSir2A). The structure of PfSir2A revealed that this sirtuin has a hydrophobic pocket that allows interaction with the acyl group of proteins [84]. The ability of sirtuins (SIRT) to remove hydrophobic acyl modifications seems to also be conserved in mammalians. SIRT1, SIRT2, SIRT3, and SIRT6 contain a hydrophobic pocket similarly to PfSir2A, indicating that the de-fatty acylation activity is maintained among different species [82,83,85]. The de-fatty acylation activity of SIRT6 has been shown to regulate tumor necrosis factor α (TNFα) secretion [83] and RAS2/phosphatidylinositol 3-kinase (PI3K) signaling [86]. These studies highlighted the role of lysine fatty acylation in the control of protein–protein interactions. Considering the nuclear localization of SIRT1 and SIRT6 [87], the emerging the de-acylating activity of sirtuins lays the foundations for a potential novel mechanism of gene expression regulation.

Accordingly, proteomic analysis in mammalian cells revealed that several variants of histone H3 were *S*-palmitoylated at cysteine 110 [88]. However, the functional role of H3 palmitoylation is still unclear, as it might modulate the stability of the H3/H4 tetramer or modify the interaction between chromatin and the nuclear membrane. In addition, Zou et al. found that, upon calcium stimulation, the enzyme acyl-CoA:lysophosphatidylcholine acyltransferase (Lpcat1) catalyzed the palmitoylation of serine 47 on histone H4 (*O*-palmitoylation) [89]. Interestingly, this modification was found to regulate RNA polymerase II activity and to activate transcription [89].

However, the effects of palmitoylation on chromatin organization are not limited to histone proteins. A recent study performed in *Saccharomyces cerevisiae* showed that palmitoyltransferase Pfa4 is implicated in heterochromatin formation via the palmitoylation of the telomere-binding protein Rif1 [90]. The telomeres, which contain domains of heterochromatin, are bound to a fibrillar network known as the nuclear lamina. The authors found that, upon palmitoylation, Rif1 reduced the silencing of chromatin by competing with Silent Information Regulator/Repressor Activator Protein 1 (SIR/RAP1) complex for telomeric DNA binding. Thereby, Rif1palmitoylation may influence telomere anchoring, nuclear organization, and heterochromatin silencing [91]. Collectively, experimental evidence indicates that protein palmitoylation can be modulated by metabolic signals and may represent a novel mechanism regulating chromatin structure and gene expression. Considering both the well-established role of aberrant palmitoylation in cancer and the emerging interest toward protein palmitoylation in neurological diseases, the following sections focus on palmitoylation targets potentially linking altered metabolic signals and human diseases.

## 6. Palmitoylation and Cancer 

Protein *S*-palmitoylation is implicated in a plethora of cellular mechanisms underlying the control of homeostasis in human physiology, and inappropriate regulation of this PTM results in the aberrant activation of different signaling cascades potentially involved in human diseases. The aberrant activation of PATs has been reported in a wide variety of human cancers [92]. Palmitoylated proteins include enzymes regulating proliferation, apoptosis, angiogenesis, and invasiveness [92]. Cross-referencing of a list of cancer driver genes with the results of palmitoylome studies suggests that 26% of the encoded proteins may be palmitoylated, highlighting the implication of this modification in cancer development [93]. The most studied palmitoylated protein, among those associated with cancer, is the guanosine triphosphate hydrolase (GTPase) RAS family [94]. Aberrant activation of the oncogene RAS, due to the defective regulation of palmitoylation, is present in up to 30% of cancer cases, resulting in uncontrolled cell growth and proliferation [95]. In vivo, anomalous processing of NRAS palmitoylation, a protein of the RAS family, is involved in leukemogenesis. Specifically, bone marrow cells transplanted in a mouse model are incapable of inducing cancer if they are transfected with the palmitoylation-deficient NRAS mutant [96]. The inhibition of NRAS palmitoylation prevents its normal membrane localization and hyper-activation of its downstream signaling effectors (Erk, Akt, Raf, and Ral) [96], demonstrating that oncogenic NRAS signaling requires palmitoylation-dependent membrane localization. The palmitoylation of both NRAS and H-RAS occurs in the Golgi compartment, and this PTM is regulated by a complex of PATs formed by zinc finger DHHC domain containing (ZDHHC)9 and Golgin (GOLG)A7 [97]. In mice lacking *Zdhhc9*, both NRAS-mediated T-cell acute lymphocytic leukemia and chronic myelomonocytic leukemia are significantly attenuated but not completely prevented. This incomplete phenotypic suppression implicates the action of multiple PATs to palmitoylate NRAS in vivo [98]. The involvement of several PATs in the control of the RAS family activity hinders the development of a pharmacology strategy against RAS-dependent cancer progression.

In recent years, it has become well known that dysregulation of the Hippo pathway results in a cancerous phenotype. Aberrant Hippo signaling is induced by mutations or altered expression levels and activity of several Hippo pathway components [99]. Equally, deactivation of the Hippo pathway impinges on cell growth and tumors [100]. A further instance of aberrant palmitoylation-dependent control over cancer-associated proteins is provided by Scribble (SCRIB), which is implicated in the Hippo pathway. SCRIB is a tumor-suppressor protein that regulates epithelial cell polarity [101,102]. Hippo kinase cascade is inactivated by the loss of SCRIB, leading to the accumulation of YAP and TAZ in the nucleus, and the activation of the mitogen-activated protein kinase (MAPK) and protein kinase B (AKT) pathways [103,104,105]. Once palmitoylated, SCRIB is stabilized in the membrane, where activates the Hippo kinase cascade and represses MAPK and AKT signaling [103,104]. DHHC7 has been identified, through co-immunoprecipitation (co-IP) experiments, as a primary SCRIB palmitoyltransferase [103]. Interestingly, knockout of DHHC7 led to a SCRIB mislocalization through the loss of palmitoylation and increased YAP nuclear localization and activation, causing a switch of SCRIB function from tumor-suppressor to oncoprotein [103]. Moreover, enhancing SCRIB palmitoylation by the inhibition of thioesterases caused an increase of SCRIB stability in the membrane, resulting in a decrease of tumorigenicity [104].

Another important palmitoylated factor involved in the Hippo pathway is TEADs, which have been found to be overexpressed in different types of tumors [106,107,108,109]. High TEAD expression levels have been observed in colorectal, breast, and prostate cancers [109]. In breast cancer cells, an upregulation of TEAD2 and a marked increase in YAP/TAZ nuclear accumulation have been observed in spite of the overall reduction of YAP/TAZ protein levels [110]. The increased expression TEAD2 resulted in an augmented binding of YAP/TAZ complex, which was retained in the nucleus leading TEAD transcriptional activity. Higher levels of TEAD2 and TEAD4 were also found in colorectal cancer, specifically in metastatic tissues, and experiments performed in vivo and in vitro have shown that knockdown of TEAD4 reduces metastasis and cell migration [109].

TEAD levels are a useful prognostic marker in cancer, and their modulation could be helpful to develop a strategy against cancer progression. In a transgenic mouse model of liver cancer mediated by YAP overexpression, transfection with a mutant TEAD lacking the DNA binding domain counteracted the YAP-induced tumor [111].

Because of the relevant roles that TEADs plays in cancer development and progression, TEAD proteins are being considered as promising therapeutic targets for antagonizing Hippo transcription under oncogenic conditions. Furthermore, a new strategy could be the development of compounds targeting the TEAD hydrophobic pocket. However, the specific acyl-transferase modulating the Hippo pathway and the target-specific effect of aberrant palmitoylation in tumor growth and invasiveness remain to be understood. 

Using both computational and experimental approaches, a recent study showed that flufenamate (a nonsteroidal anti-inflammatory drug) binds a lipid pocket and inhibits TEAD transcriptional activity without disrupting YAP–TEAD interaction, but leads to decreased cell proliferation and migration [112]. Since flufenamate binds the hydrophobic pocket of TEAD, which is the portion where palmitoylation occurs, this drug may counteract cancer development and inhibit TEAD activity by preventing its palmitoylation. 

## 7. Palmitoylation and Neurodegenerative Diseases

Palmitoylation is implicated in a series of pathologies beyond cancer, involving processes not fully understood yet. Currently, emerging evidence points to the role of aberrant palmitoylation in the onset of several neurodegenerative disorders such as Parkinson′s, Huntington (HD), and Alzheimer′s (AD) disease [113]. For instance, the dysfunction of D2 dopamine receptor (D2R), a G protein-coupled receptor (GPCR) crucial for the regulation of mood, reward, motor control, and cognition, is linked to schizophrenia and Parkinson’s disease [114,115]. Mutagenesis studies on D2R have identified Cys 443 as palmitoylation site required for plasma membrane-retention and protein stability of receptors [116]. The aberrant palmitoylation of D2R leads to dopamine dysregulation syndrome.

HD is an adult-onset neurodegenerative pathology characterized by progressive cognitive decline, motor dysfunction, and psychiatric deficit [117,118]. HD is an autosomal dominant disease caused by a mutation in the *Huntingtin* gene due to an expansion of the CAG trinucleotide repeat to more than 35 repeats, leading to an abnormal huntingtin protein (HTT) with an expanded polyglutamine in its N-terminal domain, and toxic gain of function.

HTT protein is palmitoylated at cysteine 214 (C214) by DHHC17 (also named HIP14) and DHHC13. However, upon HD mutation, the interaction between HTT and its PATs is impaired, resulting in a strong reduction of HTT palmitoylation [119,120]. It has also been demonstrated that HTT may act as a modulator of DHHC17 activity, because the activity of palmitoyl-transferase appears to be compromised upon HD mutation [121]. Consequently, PAT substrates are less palmitoylated, leading to neuronal toxicity. These evidences suggest that altered interactions between PATs and mutant HTT reduce palmitoylation and promote the mislocalization of HTT and other PAT substrates [121].

A recent study provided evidence on the involvement of palmitoylation in AD pathogenesis by proving that amyloid precursor protein (APP) is palmitoylated in vitro and in vivo, and this PTM regulates the amyloidogenic process [122]. AD is the most common form of neurodegenerative disorders characterized by the accumulation of the β-amyloid peptide (Aβ) within the brain, leading to neuronal dysfunctions, cognitive decline, and progressive memory loss [123,124]. Aβ peptide is generated by the sequential proteolysis of APP, and it is widely recognized that this is the crucial step in the development of AD [124]. APP is palmitoylated at C186 and C187 in the N-terminal region and regulates the trafficking of APP to the membrane, since palmitoylation-deficient APP mutants are retained in the endoplasmic reticulum. APP is palmitoylated by DHHC7 and DHHC21, and overexpression of this PAT leads to an increase of Aβ production as well as APP palmitoylation [122].

Furthermore, it has also been reported that the neuron-specific membrane-associated amyloid-precursor protein-cleaving enzyme 1 (BACE1) may be palmitoylated in several cysteine residues (C478, C474, C482, C485) by different PATs (DHHC3, DHHC4, DHHC7, DHHC15, and DHHC20) [125,126]. Therefore, the aberrant palmitoylation of BACE1 might promote the localization of secretase within lipid rafts and increase Aβ production [127]. Actually, APP metabolism was not affected by palmitoylation-deficient BACE1 mutants, thus indicating that BACE1 palmitoylation is not involved in Aβ production [128]. Strikingly, drugs inhibiting PAT activity decreased Aβ production, supporting the hypothesis that the palmitoylation of enzymes involved in Aβ production would be required for Aβ accumulation [129,130]. However, to date, the role of protein palmitoylation in AD onset and progression has still not been completely clarified. More importantly, aberrant palmitoylation might also influence neurodegeneration by targeting proteins other than APP, BACE1, and the canonical Aβ synthesis pathway.

For instance, estrogens have a wide range of beneficial actions in brain and other tissues, and alterations of the molecular pathways downstream from ER activation may increase the risk of AD [131]. An altered distribution of ERs has been observed in postmortem brains fromAD patients [132]. Furthermore, changes of ER localization from the nucleus to the cytoplasm inhibit the onset of AD in transgenic mice and in humans [133,134]. Studies of endogenous sex steroids depletion in wild-type female rodents have shown a significant increase of levels of soluble Aβ in the brain [135]. Furthermore, ovariectomies in numerous transgenic mouse models of AD result in the significant acceleration of Aβ pathology and the worsening of behavioral performance [136]. Moreover, female rodents treated with 17β-estradiol exhibit significantly lower Aβ accumulation as compared to females treated with a vehicle [137].

Several evidences link chromatin remodelers including sirtuins, CBP/P300, and EID1 to neurodegenerative disorders. The sirtuin pathways are strongly compromised in dementia, since a reduction of SIRT1 and SIRT3 protein levels have been observed in the AD brain. [138]. Also, in mouse models of APP and presenilin 1 overexpression, the reduction of SIRT3 has been observed [139]. The modulation of SIRT1 by activation or overexpression interferes with Aβ toxicity through its capacity to inhibit nuclear factor kappa-light-chain-enhancer of activated B cells (NF-kB) signaling [140]. Besides, CBP and P300 are mechanistically involved in the formation of amyloid-like aggregates [141] and the depletion of CBP/P300 is linked to neurodegenerative diseases [142].

SIRT1 also contributes to the pathogenesis of AD via the accumulation of the microtubule-associated protein tau. In the AD brain, tau is hyper-phosphorylated (p-tau) and forms neurofibrillary tangles. The acetylation of tau contributes to the accumulation of p-tau and the downregulation of SIRT1 increases the acetylation of tau [143] mediated by acetyltransferase p300.

Moreover, the nuclear translocation of EID1 is increased in neurons of AD brains, and EID1 overexpression in mice increments its nuclear translocation, impairing long-term potentiation (LTP) and memory. Moreover, the overexpression of EID1 results in the inhibition of CBP/p300 acetyltransferase activity and disrupts the neuronal structure, altering III-tubulin [144].

The abovementioned proteins have a role in the onset and development of neurological diseases, and, interestingly, these pathways are modulated by palmitoylation.

In recent years, an increasing number of studies have focused on the correlation between dementia and metabolic disorders such as obesity, insulin resistance, and type 2 diabetes (T2D). T2D induced the development of amyloid plaques similar to non-diabetic AD, while the pharmacological treatment of T2D reduced amyloid plaques compared to non-diabetics with similar levels of dementia [145]. Furthermore, obese patients show deficits in learning and memory as AD patients.

As discussed above, recent studies have demonstrated that diet-dependent signals may affect protein palmitoylation in different tissues, including brain areas regulating learning and memory. Moreover, restoring aberrant palmitoylation has been demonstrated to counteract brain insulin resistance-dependent cognitive decline. Based on these premises, protein palmitoylation may be a potential link between metabolic and neurodegenerative diseases. However, the lack of a specific sequence motif and the absence of a robust enrichment method hinders a more accurate understanding of palmitoylation and its regulation. Therefore, an important point is to develop high-throughput dedicated methods to study palmitoylation. The proteomic approach paired with the development of new and reliable methodologies for the recognition of palmitoylated proteins will facilitate the comprehension of the role of palmitoylation in biological functions as well as its impact on the nuclear compartment and gene regulation.

## 8. Conclusion and Future Perspectives

Emerging evidence indicates that protein palmitoylation is modulated by nutrient-dependent signals and may impact the regulation of gene expression. Palmitoylation plays a pivotal role in enzymatic activation and trafficking to the nucleus of proteins critically involved in chromatin organization. The idea that diet-derived molecules may directly change the chromatin architecture, as well as influence both gene activation and repression, opens the way to the study of novel mechanisms underlying the gene–environment interaction. Finally, aberrant palmitoylation has been linked to many human diseases including cancer, neurodegenerative diseases, and metabolic disorders [13,76,146]. Future studies revealing the impact of nutrients on protein palmitoylation will probably add novel layers to the knowledge on the link between nutrigenomics and age-related diseases.

## References

[1] Fontana L., Partridge L. (2015). Promoting health and longevity through diet: From model organisms to humans. Cell.

[2] Fusco S., Leone L., Barbati S.A., Samengo D., Piacentini R., Maulucci G., Toietta G., Spinelli M., McBurney M., Pani G. (2016). CREB-Sirt1-Hes1 circuitry mediates neural stem cell response to glucose availability. Cell Rep..

[3] Fusco S., Ripoli C., Podda M.V., Ranieri S.C., Leone L., Toietta G., McBurney M.W., Schütz G., Riccio A., Grassi C., Galeotti T. (2012). A role for neuronal cAMP responsive-element binding (CREB)-1 in brain responses to calorie restriction. Proc. Natl. Acad. Sci. USA.

[4] Mainardi M., Fusco S., Grassi C. (2015). Modulation of hippocampal neural plasticity by glucose-related signaling. Neural Plast..

[5] Hardie D.G. (2003). Minireview: The AMP-activated protein kinase cascade: The key sensor of cellular energy status. Endocrinology.

[6] Shi L., Tu B.P. (2015). Acetyl-CoA and the Regulation of Metabolism: Mechanisms and Consequences. Curr. Opin. Cell Biol..

[7] Chamberlain L.H., Shipston M.J. (2015). The physiology of protein *S*-acylation. Physiol Rev..

[8] Zhang M.M., Hang H.C. (2017). Protein S-palmitoylation in cellular differentiation. Biochem. Soc. Trans..

[9] Greaves J., Chamberlain L.H. (2011). DHHC palmitoyl transferases: Substrate interactions and (patho) physiology. Trends Biochem. Sci..

[10] Jiang H., Zhang X., Chen X., Aramsangtienchai P., Tong Z., Lin H. (2018). Protein lipidation: Occurrence, mechanisms, biological functions, and enabling technologies. Chem. Rev..

[11] Linder M.E., Deschenes R.J. (2007). Palmitoylation: Policing protein stability and traffic. Nat. Rev. Mol. Cell Biol..

[12] Salaun C., Greaves J., Chamberlain L.H. (2010). The intracellular dynamic of protein palmitoylation. J. Cell Biol..

[13] Yeste-Velasco M., Linder M.E., Lu Y.J. (2015). Protein S-palmitoylation and cancer. Biochim. Biophys. Acta.

[14] Jirtle R.L., Skinner M.K. (2007). Environmental epigenomics and disease susceptibility. Nat. Rev. Genet..

[15] Rajala R.V., Datla R.S., Moyana T.N., Kakkar R., Carlsen S.A., Sharma R.K. (2000). N-myristoyltransferase. Mol. Cell. Biochem..

[16] Johnson D.R., Bhatnagar R.S., Knoll L.J., Gordon J.I. (1994). Genetic and biochemical studies of protein N-myristoylation. Annu Rev. Biochem.

[17] Thinon E., Serwa R.A., Broncel M., Brannigan J.A., Brassat U., Wright M.H., Heal W.P., Wilkinson A.J., Mann D.J., Tate E.W. (2014). Global profiling of co- and post-translationally N-myristoylated proteomes in human cells. Nat. Commun..

[18] Furfine E.S., Leban J.J., Landavazo A., Moomaw J.F., Casey P.J. (1995). Protein farnesyltransferase: Kinetics of farnesyl pyrophosphate binding and product release. Biochemistry.

[19] Moores S.L., Schaber M.D., Mosser S.D., Rands E., O’Hara M.B., Garsky V.M., Marshall M.S., Pompliano D.L., Gibbs J. (1991). Sequence dependence of protein isoprenylation. J. Biol. Chem..

[20] Nalivaeva N.N., Turner A.J. (2001). Post-translational modifications of proteins: Acetylcholinesterase as a model system. Proteomics.

[21] Wang M., Casey P.J. (2016). Protein prenylation: Unique fats make their mark on biology. Nat. Rev. Mol. Cell Biol..

[22] Palsuledesai C.C., Distefano M.D. (2015). Protein prenylation: Enzymes, therapeutics, and biotechnology applications. ACS Chem. Biol..

[23] Linder M.E., Deschenes R.J. (2004). Model organisms lead the way to protein palmitoyltransferases. J. Cell Sci..

[24] Sanders S.S., Martin D.D., Butland S.L., Lavallée-Adam M., Calzolari D., Kay C., Yates J.R., Hayden M.R. (2015). Curation of the mammalian palmitoylome indicates a pivotal role for palmitoylation in diseases and disorders of the nervous system and cancers. PLoS Comput. Biol..

[25] Xu Z., Zhong L. (2015). New insights into the posttranslational regulation of human cytosolic thioredoxin by S-palmitoylation. Biochem. Biophys. Res. Commun..

[26] Spinelli M., Fusco S., Mainardi M., Scala F., Natale F., Lapenta R., Mattera A., Rinaudo M., Li Puma D.D., Ripoli C. (2017). Brain insulin resistance impairs hippocampal synaptic plasticity and memory by increasing GluA1 palmitoylation through FoxO3a. Nat. Commun..

[27] Mitchell D.A., Mitchell G., Ling Y., Budde C., Deschenes R.J. (2010). Mutational analysis of Saccharomyces cerevisiae Erf2 reveals a two-step reaction mechanism for protein palmitoylation by DHHC enzymes. J. Biol. Chem..

[28] Yousefi-Salakdeh E., Johansson J., Strömberg R. (1999). A method for S- and O-palmitoylation of peptides: Synthesis of pulmonary surfactant protein-C. models. Biochem. J..

[29] Buglino J.A., Resh M.D. (2008). Hhat is a palmitoylacyltransferase with specificity for N-palmitoylation of Sonic Hedgehog. J. Biol. Chem..

[30] Ji Y., Bachschmid M.M., Costello C.E., Lin C. (2016). S- to N-Palmitoyl Transfer During Proteomic Sample Preparation. J. Am. Soc. Mass Spectrom..

[31] Hackett M., Guo L., Shabanowitz J., Hunt D.F., Hewlett E.L. (1994). Internal lysine palmitoylation in adenylate cyclase toxin from Bordetella pertussis. Science.

[32] Mitchell D.A., Vasudevan A., Linder M.E., Deschenes R.J. (2006). Protein palmitoylation by a family of DHHC protein S.-acyltransferases. J. Lipid Res..

[33] Fukata M., Fukata Y., Adesnik H., Nicoll R.A., Bredt D.S. (2004). Identification of PSD-95 palmitoylating enzymes. Neuron.

[34] Lobo S., Greentree W.K., Linder M.E., Deschenes R.J. (2002). Identification of a Ras palmitoyltransferase in Saccharomyces cerevisiae. J. Biol. Chem..

[35] Roth A.F., Feng Y., Chen L., Davis N.G. (2002). The yeast DHHC cysteine-rich domain protein Akr1p is a palmitoyl transferase. J. Cell Biol..

[36] Yang W., Di Vizio D., Kirchner M., Steen H., Freeman M.R., Proteomics C. (2010). Proteome scale characterization of human S-acylated proteins in lipid raft-enriched and non-raft membranes. Mol. Cell. Proteomics.

[37] Jennings B.C., Linder M.E. (2012). DHHC protein S-acyltransferases use a similar ping-pong kinetic mechanism but display different acyl-coA specificities. J. Biol. Chem..

[38] Duncan J.A., Gilman A.G. (2002). Characterization of Saccharomyces cerevisiaeAcyl-protein Thioesterase 1, the Enzyme Responsible for G Protein α Subunit Deacylation in Vivo. J. Biol. Chem..

[39] Kong E., Peng S., Chandra G., Sarkar C., Zhang Z., Bagh M.B., Mukherjee A.B. (2013). Dynamic palmitoylation links cytosol-membrane shuttling of acyl-protein thioesterase-1 and acyl-protein thioesterase-2 with that of proto-oncogene H-Ras product and growth associated protein-43. J. Biol. Chem..

[40] Lin D.T., Conibear E. (2015). ABHD17 proteins are novel protein depalmitoylases that regulate N-Ras palmitate turnover and subcellular localization. eLife.

[41] Grammel M., Hang H.C. (2013). Chemical reporters for biological discovery. Nat. Chem. Biol..

[42] Thiele C., Papan C., Hoelper D., Kusserow K., Gaebler A., Schoene M., Piotrowitz K., Lohmann D., Spandl J., Stevanovic A. (2012). Tracing fatty acid metabolism by click chemistry. ACS Chem. Biol..

[43] Zhang M.M., Tsou L.K., Charron G., Raghavan A.S., Hang H.C. (2010). Tandem fluorescence imaging of dynamic S-acylation and protein turnover. Proc. Natl. Acad. Sci. USA.

[44] El-Husseini A.D., Schnell E., Dakoji S., Sweeney N., Zhou Q., Prange O., Gauthier-Campbell C., Aguilera-Moreno A., Nicoll R.A., Bredt D.S. (2002). Synaptic strength regulated by palmitate cycling on PSD-95. Cell.

[45] Dekker F.J., Rocks O., Vartak N., Menninger S., Hedberg C., Balamurugan R., Wetzel S., Renner S., Gerauer M., Schölermann B. (2010). Small-molecule inhibition of APT1 affects Ras localization and signaling. Nat. Chem. Biol..

[46] Kang R., Wan J., Arstikaitis P., Takahashi H., Huang K., Bailey A.O., Thompson J.X., Roth A.F., Drisdel R.C., Mastro R. (2008). Neural palmitoyl-proteomics reveals dynamic synaptic palmitoylation. Nature.

[47] Leto D., Saltiel A.R. (2012). Regulation of glucose transport by insulin: Traffic control of GLUT4. Nat. Rev. Mol. Cell Biol..

[48] Chang L., Chiang S.H., Saltiel A.R. (2004). Insulin signaling and the regulation of glucose transport. Mol. Med..

[49] Thorens B., Mueckler M. (2010). Metabolism, Glucose transporters in the 21st Century. Am. J. Physiol. Endocrinol. Metab..

[50] Ren W., Sun Y., Du K. (2015). Glut4 palmitoylation at Cys223 plays a critical role in Glut4 membrane trafficking. Biochem. Biophys. Res. Commun..

[51] Du K., Murakami S., Sun Y., Kilpatrick C., Luscher B. (2017). DHHC7 palmitoylates Glut4 and regulates Glut4 membrane translocation. J. Biol. Chem..

[52] Wei X., Song H., Semenkovich C.F. (2014). Insulin-Regulated Protein Palmitoylation Impacts Endothelial Cell Function. Arterioscler. Thromb. Vasc. Biol..

[53] Ren W., Jhala U.S., Du K. (2013). Proteomic analysis of protein palmitoylation in adipocytes. Adipocyte.

[54] Kathayat R.S., Cao Y., Elvira P.D., Sandoz P.A., Zaballa M.-E., Springer M.Z., Drake L.E., Macleod K.F., Van der Goot F.G., Dickinson B.C. (2018). Active and dynamic mitochondrial S-depalmitoylation revealed by targeted fluorescent probes. Nat. Commun..

[55] Tao N., Wagner S.J., Lublin D.M. (1996). CD36 is palmitoylated on both N- and C-terminal cytoplasmic tails. J. Biol. Chem..

[56] Zhao L., Zhang C., Luo X., Wang P., Zhou W., Zhong S., Xie Y., Jiang Y., Yang P., Tang R. (2018). CD36 palmitoylation disrupts free fatty acid metabolism and promotes tissue inflammation in non-alcoholic steatohepatitis. J. Hepatol..

[57] Yamada S., Komatsu M., Sato Y., Yamauchi K., Aizawa T., Kojima I. (2003). Nutrient modulation of palmitoylated 24-kilodalton protein in rat pancreatic islets. Endocrinology.

[58] Abdel-Ghany M., Sharp G.W., Straub S.G. (2010). Glucose stimulation of protein acylation in the pancreatic β-cell. Life Sci..

[59] Tobin A.B., Wheatley M. (2004). G-protein-coupled receptor phosphorylation and palmitoylation. Met. Mol. Biol..

[60] Gubitosi-Klug R.A., Mancuso D.J., Gross R.W. (2005). The human Kv1.1 channel is palmitoylated modulating voltage sensing. Identification of a palmitoylation concensus sequence. Proc. Natl. Acad. Sci. USA.

[61] Hurley J.H., Cahill A.L., Currie K.P., Fox A.P. (2000). The role of dynamic palmitoylation in Ca^2+^ channel inactivation. Proc. Natl. Acad. Sci. USA.

[62] Roth A.F., Wan J., Bailey A.O., Sun B., Kuchar J.A., Green W.N., Phinney B.S., Yates J.R., Davis N.G. (2006). Global analysis of protein palmitoylation in yeast. Cell.

[63] Cao Y., Huang Y. (2005). Palmitoylation regulates GDP/GTP exchange of G protein by affecting the GTP-binding activity of Goα. Int. J. Biochem. Cell Biol..

[64] Levin E.R. (2011). Minireview: Extranuclear steroid receptors: Roles in modulation of cell functions. Mol. Endocrinol..

[65] Pedram A., Razandi M., Sainson R.C., Kim J.K., Hughes C.C., Levin E.R. (2007). A conserved mechanism for steroid receptor translocation to the plasma membrane. J. Biol. Chem..

[66] Razandi M., Alton G., Pedram A., Ghonshani S., Webb P., Levin E.R. (2003). Identification of a structural determinant necessary for the localization and function of estrogen receptor α at the plasma membrane. Mol. Cell. Biol..

[67] Acconcia F., Ascenzi P., Bocedi A., Spisni E., Tomasi V., Trentalance A., Visca P., Marino M. (2005). Palmitoylation-dependent Estrogen Receptor α Membrane Localization: Regulation by 17β-Estradiol. Mol. Biol. Cell.

[68] Acconcia F., Ascenzi P., Fabozzi G., Visca P., Marino M. (2004). S-palmitoylation modulates human estrogen receptor-α functions. Biochem. Biophys. Res. Commun..

[69] Adlanmerini M., Solinhac R., Abot A., Fabre A., Raymond-Letron I., Guihot A.-L., Boudou F., Sautier L., Vessières E., Kim S.H. (2014). Mutation of the palmitoylation site of estrogen receptor α in vivo reveals tissue-specific roles for membrane versus nuclear actions. Proc. Natl. Acad. Sci. USA.

[70] Chan P., Han X., Zheng B., DeRan M., Yu J., Jarugumilli G.K., Deng H., Pan D., Luo X., Wu X. (2016). Autopalmitoylation of TEAD proteins regulates transcriptional output of the Hippo pathway. Nat. Chem. Biol..

[71] Noland C.L., Gierke S., Schnier P.D., Murray J., Sandoval W.N., Sagolla M., Dey A., Hannoush R.N., Fairbrother W.J., Cunningham C.N. (2016). Palmitoylation of TEAD transcription factors is required for their stability and function in Hippo pathway signaling. Structure.

[72] Lamar J.M., Stern P., Liu H., Schindler J.W., Jiang Z.G., Hynes R.O. (2012). The hippo pathway target, YAP, promotes metastasis through its TEAD-interaction domain. Proc. Natl. Acad. Sci. USA.

[73] Benayoun B.A., Veitia R.A. (2009). A post-translational modification code for transcription factors: Sorting through a sea of signals. Trends Cell Biol..

[74] Duan M., Zhang R., Zhu F., Zhang Z., Gou L., Wen J., Dong J., Wang T. (2017). A Lipid-Anchored NAC Transcription Factor Is Translocated into the Nucleus and Activates Glyoxalase I Expression during Drought Stress. Plant Cell.

[75] Eisenhaber B., Sammer M., Lua W.H., Benetka W., Liew L.L., Yu W., Lee H.K., Koranda M., Eisenhaber F., Adhikari S. (2011). Nuclear import of a lipid-modified transcription factor: Mobilization of NFAT5 isoform a by osmotic stress. Cell Cycle.

[76] Aaron C., Baldwin C.D., Green L., Olson K., Moxley M.A., Corbett J.A. (2012). A role for aberrant protein palmitoylation in FFA-induced ER stress and β-cell death. Am. J. Physiol. Endocrinol. Metab..

[77] Riccio A. (2010). Dynamic epigenetic regulation in neurons: Enzymes, stimuli and signaling pathways. Nat. Neurosci..

[78] Misteli T., Soutoglou E. (2009). The emerging role of nuclear architecture in DNA repair and genome maintenance. Nat. Rev. Mol. Cell Biol..

[79] Bannister A.J., Kouzarides T. (2011). Regulation of chromatin by histone modifications. Cell Res..

[80] Chen X., Du Z., Shi W., Wang C., Yang Y., Wang F., Yao Y., He K., Hao A. (2014). 2-Bromopalmitate modulates neuronal differentiation through the regulation of histone acetylation. Stem Cell Res..

[81] Chen X., Du Z., Li X., Wang L., Wang F., Shi W., Hao A. (2016). Protein palmitoylation regulates neural stem cell differentiation by modulation of EID1 activity. Mol. Neurobiol..

[82] Feldman J.L., Baeza J., Denu J.M. (2013). Activation of the protein deacetylase SIRT6 by long-chain fatty acids and widespread deacylation by mammalian sirtuins. J. Biol. Chem..

[83] Jiang H., Khan S., Wang Y., Charron G., He B., Sebastian C., Du J., Kim R., Ge E., Mostoslavsky R. (2013). SIRT6 regulates TNF-α secretion through hydrolysis of long-chain fatty acyl lysine. Nature.

[84] Zhu A.Y., Zhou Y., Khan S., Deitsch K.W., Hao Q., Lin H. (2012). Plasmodium falciparum Sir2A preferentially hydrolyzes medium and long chain fatty acyl lysine. ACS Chem. Biol..

[85] Teng Y.-B., Jing H., Aramsangtienchai P., He B., Khan S., Hu J., Lin H., Hao Q. (2015). Efficient demyristoylase activity of SIRT2 revealed by kinetic and structural studies. Sci. Rep..

[86] Zhang X., Spiegelman N.A., Nelson O.D., Jing H., Lin H. (2017). SIRT6 regulates Ras-related protein R-Ras2 by lysine defatty-acylation. eLife.

[87] Michan S., Sinclair D. (2007). Sirtuins in mammals: Insights into their biological function. Biochem. J..

[88] Wilson J.P., Raghavan A.S., Yang Y.-Y., Charron G., Hang H.C., Proteomics C. (2011). Proteomic analysis of fatty-acylated proteins in mammalian cells with chemical reporters reveals S-acylation of histone H3 variants. Mol. Cell. Proteom..

[89] Zou C., Ellis B.M., Smith R.M., Chen B.B., Zhao Y., Mallampalli R.K. (2011). Acyl-CoA: Lysophosphatidylcholine acyltransferase I (Lpcat1) catalyzes histone protein O-palmitoylation to regulate mRNA synthesis. J. Biol. Chem..

[90] Park S., Patterson E.E., Cobb J., Audhya A., Gartenberg M.R., Fox C.A. (2011). Palmitoylation controls the dynamics of budding-yeast heterochromatin via the telomere-binding protein Rif1. Proc. Natl. Acad. Sci. USA.

[91] Honda T., Soeda S., Tsuda K., Yamaguchi C., Aoyama K., Morinaga T., Yuki R., Nakayama Y., Yamaguchi N., Yamaguchi N. (2016). Protective role for lipid modifications of Src-family kinases against chromosome missegregation. Sci. Rep..

[92] Ko P.J., Dixon S.J. (2018). Protein palmitoylation and cancer. EMBO Rep..

[93] Bailey M.H., Tokheim C., Porta-Pardo E., Sengupta S., Bertrand D., Weeras- inghe A., Colaprico A., Wendl M.C., Kim J., Reardon B. (2018). Comprehensive characterization of cancer driver genes and mutations. Cell.

[94] Schmick M., Kraemer A., Bastiaens P.I.H. (2015). Ras moves to stay in place. Trends Cell Biol..

[95] Eisenberg S., Laude A.J., Beckett A.J., Mageean C.J., Aran V., Hernandez-Valladares M., Henis Y.I., Prior I.A. (2013). The role of palmitoylation in regulating Ras localization and function. Biochem. Soc. Trans..

[96] Cuiffo B., Ren R. (2010). Palmitoylation of oncogenic NRAS is essential for leukemogenesis. Blood.

[97] Young E., Zheng Z.Y., Wilkins A.D., Jeong H.T., Li M., Lichtarge O., Chang E.C. (2014). Regulation of Ras localization and cell transformation by evolutionarily conserved palmitoyltransferases. Mol. Cell Biol..

[98] Liu P., Jiao B., Zhang R., Zhao H., Zhang C., Wu M., Li D., Zhao X., Qiu Q., Li J. (2016). Palmitoylacyltransferase Zdhhc9 inactivation mitigates leukemogenic potential of oncogenic Nras. Leukemia.

[99] Zhang X., Zhao H., Li Y., Xia D., Yang L., Ma Y., Li H. (2018). The role of YAP/TAZ activity in cancer metabolic reprogramming. Mol. Cancer.

[100] Overholtzer M., Zhang J., Smolen G.A., Muir B., Li W., Sgroi D.C., Deng C.X., Brugge J.S., Haber D.A. (2006). Transforming properties of YAP, a candidate oncogene on the chromosome 11q22 amplicon. Proc. Natl. Acad. Sci. USA.

[101] Pearson H.B., Perez-Mancera P.A., Dow L.E., Ryan A., Tennstedt P., Bogani D., Elsum I., Greenfield A., Tuveson D.A., Simon R. (2011). SCRIB expression is deregulated in human prostate cancer, and its deficiency in mice promotes prostate neoplasia. J. Clin. Investig..

[102] Zhan L., Rosenberg A., Bergami K.C., Yu M., Xuan Z., Jaffe A.B., Allred C., Muthuswamy S.K. (2008). Deregulation of scribble promotes mammary tumorigenesis and reveals a role for cell polarity in carcinoma. Cell.

[103] Chen B., Zheng B., DeRan M., Jarugumilli G.K., Fu J., Brooks Y.S., Wu X. (2016). ZDHHC7-mediated S-palmitoylation of Scribble regulates cell polarity. Nat. Chem. Biol..

[104] Hernandez J.L., Davda D., Cheung See Kit M., Majmudar J.D., Won S.J., Gang M., Pasupuleti S.C., Choi A.I., Bartkowiak C.M., Martin B.R. (2017). APT2 Inhibition Restores Scribble Localization and S-Palmitoylation in Snail-Transformed Cells. Cell Chem. Biol..

[105] Johnson R., Halder G. (2014). The two faces of Hippo: Targeting the Hippo pathway for regenerative medicine and cancer treatment. Nat. Rev. Drug Discov..

[106] Zhou Y., Huang T., Cheng A.S., Yu J., Kang W., To K.F. (2016). The TEAD Family and Its Oncogenic Role in Promoting Tumorigenesis. Int. J. Mol. Sci..

[107] Lim B., Park J.L., Kim H.J., Park Y.K., Kim J.H., Sohn H.A., Noh S.M., Song K.S., Kim W.H., Kim Y.S. (2014). Integrative genomics analysis reveals the multilevel dysregulation and oncogenic characteristics of TEAD4 in gastric cancer. Carcinogenesis.

[108] Yu M.H., Zhang W. (2016). TEAD1 enhances proliferation via activating SP1 in colorectal cancer. Biomed. Pharmacother..

[109] Liu Y., Wang G., Yang Y., Mei Z., Liang Z., Cui A., Wu T., Liu C.Y., Cui L. (2016). Increased TEAD4 expression and nuclear localization in colorectal cancer promote epithelial-mesenchymal transition and metastasis in a YAP-independent manner. Oncogene.

[110] Diepenbruck M., Waldmeier L., Ivanek R., Berninger P., Arnold P., van Nimwegen E., Christofori G. (2014). Tead2 expression levels control the subcellular distribution of Yap and Taz, zyxin expression and epithelial-mesenchymal transition. J. Cell Sci..

[111] Liu-Chittenden Y., Huang B., Shim J.S., Chen Q., Lee S.J., Anders R.A., Liu J.O., Pan D. (2012). Genetic and pharmacological disruption of the TEAD-YAP complex suppresses the oncogenic activity of YAP. Genes Dev..

[112] Pobbati A.V., Han X., Hung A.W., Weiguang S., Huda N., Chen G.Y., Kang C., Chia C.S., Luo X., Hong W. (2015). Targeting the Central Pocket in Human Transcription Factor TEAD as a Potential Cancer Therapeutic Strategy. Structure.

[113] Zaręba-Kozioł M., Figiel I., Bartkowiak-Kaczmarek A., Włodarczyk J. (2018). Insights into Protein S-Palmitoylation in Synaptic Plasticity and Neurological Disorders: Potential and Limitations of Methods for Detection and Analysis. Front. Mol. Neurosci..

[114] Olanow C.W., Obeso J.A., Stocchi F. (2006). Continuous dopamine-receptor treatment of Parkinson’s disease: Scientific rationale and clinical implications. Lancet Neurol..

[115] Fredericks D., Norton J.C., Atchison C., Schoenhaus R., Pill M.W. (2017). Parkinson’s disease and Parkinson’s disease psychosis: A perspective on the challenges, treatments, and economic burden. Am. J. Manag. Care.

[116] Ebersole B., Petko J., Woll M., Murakami S., Sokolina K., Wong V., Stagljar I., Lüscher B., Levenson R. (2015). Effect of C-Terminal S-Palmitoylation on D2 Dopamine Receptor Trafficking and Stability. PLoS ONE.

[117] Bates G.P., Dorsey R., Gusella J.F., Hayden M.R., Kay C., Leavitt B.R., Nance M., Ross C.A., Scahill R.I., Wetzel R. (2015). Huntington disease. Nat. Rev. Dis. Primers..

[118] Dayalu P., Albin R.L. (2015). Huntington disease: Pathogenesis and treatment. Neurol. Clin..

[119] Milnerwood A.J., Parsons M.P., Young F.B., Singaraja R.R., Franciosi S., Volta M., Bergeron S., Hayden M.R., Raymond L.A. (2013). Memory and synaptic deficits in Hip14/DHHC17 knockout mice. Proc. Natl. Acad. Sci. USA.

[120] Gottlieb C.D., Zhang S., Linder M.E. (2015). The Cysteine-rich Domain of the DHHC3 Palmitoyltransferase Is Palmitoylated and Contains Tightly Bound Zinc. J. Biol. Chem..

[121] Verardi R., Kim J.S., Ghirlando R., Banerjee A. (2017). Structural Basis for Substrate Recognition by the Ankyrin Repeat Domain of Human DHHC17 Palmitoyltransferase. Structure.

[122] Bhattacharyya R., Barren C., Kovacs D.M. (2013). Palmitoylation of amyloid precursor protein regulates amyloidogenic processing in lipid rafts. J. Neurosci..

[123] Karch C.M., Goate A.M. (2015). Alzheimer’s disease risk genes and mechanisms of disease pathogenesis. Biol. Psychiatry.

[124] Selkoe D.J., Hardy J. (2016). The amyloid hypothesis of Alzheimer’s disease at 25 years. EMBO Mol. Med..

[125] Vetrivel K.S., Meckler X., Chen Y., Nguyen P.D., Seidah N.G., Vassar R., Wong P.C., Fukata M., Kounnas M.Z., Thinakaran G. (2009). Alzheimer disease Abeta production in the absence of S-palmitoylation-dependent targeting of BACE1 to lipid rafts. J. Biol. Chem..

[126] Benjannet S., Elagoz A., Wickham L., Mamarbachi M., Munzer J.S., Basak A., Lazure C., Cromlish J.A., Sisodia S., Checler F. (2001). Post-translational processing of beta-secretase (beta-amyloid-converting enzyme) and its ectodomain shedding. The pro- and transmembrane/cytosolic domains affect its cellular activity and amyloid-beta production. J. Biol. Chem..

[127] Vetrivel K.S., Thinakaran G. (2010). Membrane rafts in Alzheimer’s disease beta-amyloid production. Biochim. Biophys. Acta.

[128] Motoki K., Kume H., Oda A., Tamaoka A., Hosaka A., Kametani F., Araki W. (2012). Neuronal β-amyloid generation is independent of lipid raft association of β-secretase BACE1: Analysis with a palmitoylation-deficient mutant. Brain Behav..

[129] Sidera C., Parsons R., Austen B. (2004). Proteolytic cascade in the amyloidogenesis of Alzheimer’s disease. Biochem. Soc. Trans..

[130] Parsons R.B., Austen B.M. (2007). Protein-protein interactions in the assembly and subcellular trafficking of the BACE (beta-site amyloid precursor protein-cleaving enzyme) complex of Alzheimer’s disease. Biochem. Soc. Trans..

[131] Liu X.-A., Zhu L.-Q., Zhang Q., Shi H.-R., Wang S.-H., Wang Q., Wang J.Z. (2008). Estradiol attenuates tau hyperphosphorylation induced by upregulation of protein kinase-A. Neurochem. Res..

[132] Yue X., Lu M., Lancaster T., Cao P., Honda S.-I., Staufenbiel M., Harada N., Zhong Z., Shen Y., Li R. (2005). Brain estrogen deficiency accelerates Aβ plaque formation in an Alzheimer’s disease animal model. Proc. Natl. Acad. Sci. USA.

[133] Jorm A., Korten A., Henderson A.S. (1987). The prevalence of dementia: A quantitative integration of the literature. Acta Psychiatr. Scand..

[134] Henderson V.W., Paganini-Hill A., Emanuel C.K., Dunn M.E., Buckwalter J.G. (1994). Estrogen replacement therapy in older women: Comparisons between Alzheimer’s disease cases and nondemented control subjects. Arch. Neurol..

[135] Petanceska S.S., Nagy V., Frail D., Gandy S. (2000). Ovariectomy and 17β-estradiol modulate the levels of Alzheimer’s amyloid β peptides in brain. Neurology.

[136] Carroll J.C., Rosario E.R., Chang L., Stanczyk F.Z., Oddo S., LaFerla F.M., Pike C.J. (2007). Progesterone and estrogen regulate Alzheimer-like neuropathology in female 3xTg-AD mice. J. Neurosci..

[137] Zhao L., Yao J., Mao Z., Chen S., Wang Y., Brinton R.D. (2011). 17β-Estradiol regulates insulin-degrading enzyme expression via an ERβ/PI3-K pathway in hippocampus: Relevance to Alzheimer’s prevention. Neurobiol. Aging.

[138] Lutz M.I., Milenkovic I., Regelsberger G., Kovacs G.G. (2014). Distinct patterns of sirtuin expression during progression of Alzheimer’s disease. Neuromol. Med..

[139] Yang W., Zou Y., Zhang M., Zhao N., Tian Q., Gu M., Liu W., Shi R., Lü Y., Yu W. (2015). Mitochondrial Sirt3 expression is decreased in APP/PS1 double transgenic mouse model of Alzheimer’s disease. Neurochem. Res..

[140] Chen J., Zhou Y., Mueller-Steiner S., Chen L.-F., Kwon H., Yi S., Mucke L., Gan L. (2005). SIRT1 protects against microglia-dependent amyloid-β toxicity through inhibiting NF-κB signaling. J. Biol. Chem..

[141] Olzscha H., Fedorov O., Kessler B.M., Knapp S., La Thangue N.B. (2017). CBP/p300 bromodomains regulate amyloid-like protein aggregation upon aberrant lysine acetylation. Cell Chem. Biol..

[142] Valor M.L., Viosca J., Lopez-Atalaya P.J., Barco A. (2013). Lysine acetyltransferases CBP and p300 as therapeutic targets in cognitive and neurodegenerative disorders. Curr. Pharm. Des..

[143] Min S.-W., Cho S.-H., Zhou Y., Schroeder S., Haroutunian V., Seeley W.W., Huang E.J., Shen Y., Masliah E., Mukherjee C. (2010). Acetylation of tau inhibits its degradation and contributes to tauopathy. Neuron.

[144] Liu R., Lei J.X., Luo C., Lan X., Chi L., Deng P., Lei S., Ghribi O., Liu Q.Y. (2012). Increased EID1 nuclear translocation impairs synaptic plasticity and memory function associated with pathogenesis of Alzheimer’s disease. Neurobiol. Dis..

[145] Beeri M., Schmeidler J., Silverman J., Gandy S., Wysocki M., Hannigan C., Purohit D., Lesser G., Grossman H., Haroutunian V. (2008). Insulin in combination with other diabetes medication is associated with less Alzheimer neuropathology. Neurology.

[146] Cho E., Park M. (2016). Palmitoylation in Alzheimer’s disease and other neurodegenerative diseases. Pharmacol. Res..

